# Refugee Caregivers’ Perceptions of Using Mindfulness-Based Interventions to Support Coping Skills in Children with Disability in Jordan

**DOI:** 10.3390/children11111381

**Published:** 2024-11-13

**Authors:** Zeina Fayad, Hadeel R. Bakhsh, Rawan AlHeresh

**Affiliations:** 1 Department of Occupational Therapy, The Lab School of Washington, Washington, DC 20007, USA; zeina.fayad@labschool.org; 2 Department of Rehabilitation Sciences, College of Health and Rehabilitation Sciences, Princess Nourah Bint Abdulrahman University, Riyadh 11564, Saudi Arabia; 3 Department of Occupational Therapy, Faculty of Rehabilitation Sciences, University of Jordan, Queen Rania St., Amman 11942, Jordan; r.alheresh@ju.edu.jo

**Keywords:** children with disabilities, child refugees, mindfulness, mental health, community-based rehabilitation, caregivers

## Abstract

**Background:** Refugee children with disabilities are vulnerable to developing dysfunctional coping skills due to barriers in accessing health care services, including occupational therapy (OT). This study investigated the perceptions of refugee caregivers regarding the use of mindfulness-based interventions (MBIs) as a treatment for coping skills among their children with disabilities. **Methods**: A qualitative survey was used to analyse the coping mechanisms and access to services among refugee children with disabilities, as well as their caregivers’ perceptions regarding the use of MBIs. The caregivers were recruited using convenience sampling from a database of individuals at the Al-Baqa’a refugee camp in Jordan via their community-based rehabilitation (CBR) centre. A demographic survey was sent to 156 refugees using WhatsApp. Refugees above the age of 18 years identifying as the caregivers of children with disabilities were eligible. Twenty-eight individuals completed the survey, and twenty-six were eligible. Open-ended questions asked about their child’s struggles, supports, barriers to support, and perceptions of MBIs. The data were collected via Google Sheets. Three researchers coded the responses using a descriptive coding method and line-by-line analysis. **Results**: Five themes were derived from the responses: “Daily Challenges”, “Support and Strategies”, “barriers to accessing support”, “MBIs: Caregiver Perceptions”, and “MBIs: Barriers”. The participants identified the behavioural, emotional, and cognitive challenges impacting their child’s participation. Financial and environmental constraints, stigma, and timing were the reported barriers to services. The participants felt positive about the potential of MBIs to support their children. **Conclusions**: Overall, this study obtained foundational data to establish accessible mental health programmes for refugees with disabilities. Further research is needed to transcend the barriers and maintain MBI programmes in the community and the home settings.

## 1. Introduction

The United Nations has defined the ongoing refugee crisis as the “biggest humanitarian emergency in our era” [1]. Jordan currently houses over 2 million Palestinian refugees and 67,300 Syrian refugees in 15 camps located around the country [2,3], with more than 50% of the Syrian refugees being children under the age of 18 years [4]. This places an immense strain on financial and medical infrastructure in trying to meet the refugees’ extensive healthcare needs, in addition to supporting other citizens [5]. Jordan hosts the highest number of child refugees in the world relative to their population [6]. Migration is a difficult event, as the loss of one’s accustomed environment and support systems can intensify or cause new mental issues. Refugees may also endure prejudice, marginalisation, and social exclusion in their host communities, which can have a negative impact on their mental health and wellbeing. The World Health Organization has called for external support, further research, and increased mental health aid to support the needs of children in Jordan [6], refugees and native citizens alike [7].

Several other studies demonstrated that refugee children and adolescents are the most severely affected by psychosocial problems, like post-traumatic stress syndrome (PTSD), panic attacks, sleep disorders, depression, suicidality, and others [8,9,10,11,12,13]. There is a lack of mental health care accessibility in Jordan, which is a salient barrier for child refugees to receiving the appropriate services. Although families flee from perilous environments and war to find safety, the experience of living in a new country also has challenges. Khamis et al. found that 46.6% of Syrian refugee children in Lebanon and Jordan have PTSD, with a high risk of developing emotional dysregulation, and this was largely reported by older refugee children and adolescents [14,15]. Consequently, the stress of resettlement and trauma overwhelm a child’s ability to cope, which can result in the development of disruptive coping strategies and long-term psychosocial dysfunction and difficulties in sleeping, mood swings, isolation, grief, and lower activity levels [16].

Refugee children with disabilities are at a greater disadvantage in accessing support. Refugee children with disabilities are largely excluded from social programmes that support healthy development and learning due to limited or non-existent resources, as well as discrimination [17,18]. Promoting inclusion and fair access to services among child refugees with disabilities should be a priority, as they are at a higher risk of experiencing exploitation, abuse, violence, and toxicity without receiving appropriate support [19,20]. Such neglect will lead to the exacerbation of existing conditions, as well as the onset of mental health conditions and negative coping patterns [21]. Therefore, this study will focus on this subpopulation for these reasons.

Coping is defined as the use of cognitive and behavioural means to process and internally synthesise stressful demands to maintain functioning in daily life [22]. The absence of positive and active coping skills can harm a child’s wellbeing, significantly altering the course of their life [22,23]. Therefore, the lack of mental health programmes among refugee children diminishes the use of positive coping skills and decreases the potential for positive life outcomes [23,24].

Mental health programmes support the development of coping strategies and increase the overall symptom management and quality of life of refugee children [23]. There is a gap in the literature supporting mental health initiatives for refugee children with disabilities and a pressing need for increased research. To overcome the existing barriers and establish sustainable initiatives, the mental health programmes for refugee children with disabilities must be cost-effective, accessible, and culturally appropriate [5,23]. Therefore, it is suggested that the first level of mental health intervention response be a trauma-informed intervention administered in group settings or community centres to increase feelings of belongingness and comfort [25]; an example of such an intervention includes mind–body connectedness and mindfulness [23]. Mindfulness-based interventions (MBIs) refer to the therapeutic strategies used to nurture coping skills, such as meditation, yoga, arts and crafts, and physical movement [26]. MBIs are considered to promote non-judgmental thoughts, mind–body connections, and increased awareness, all of which enable the development of self-compassion and coping skills in children [27,28,29]. Moreover, MBIs support social participation, educational engagement, prosocial behaviours, and cognitive flexibility by improving cognitive functioning and self-regulation skills [30,31,32].

There is limited research on the use of MBIs with refugee children in the Middle East, although this is an emerging topic with promising results. One study demonstrated the positive effects of MBIs on Middle Eastern refugees as a way to mitigate the effects of depressive symptoms on daily living [21]. Another study among Syrian refugee children in Turkey promoted the use of group psychosocial interventions in classroom settings, which is an important precursor to the use of MBIs among refugee child communities [26]. For instance, a dance MBI was found to decrease anxiety symptoms, increase socialisation, and promote school attendance among Syrian refugees in the US [33,34]. Furthermore, a mindfulness programme for unaccompanied refugee youth showed a decrease in depressive symptoms and negative effects [35]. Therefore, MBIs have been shown to be trauma-informed and accessible and an appropriate foundation for mental health programming among refugee children in Jordan.

While there is a wide variety of literature that supports the use of MBIs to address social emotional, mental health, and cognitive challenges in children with disabilities [36,37,38,39], there is currently no evidence to support these trends in the subpopulation of child refugees with disabilities. This study aimed to investigate the use of mindfulness-based interventions (MBIs) as feasible, community-based, and accessible mental health programmes for refugee children with disabilities. In order to do so, the current study will investigate refugee caregivers’ perceptions of using MBIs as a means to develop coping strategies among their disabled children by analysing their survey responses.

The research questions addressed in this paper are as follows: What are daily barriers and support options to accessing healthcare among refugee children with disability? What are refugee caregivers’ perceptions of using a mindfulness-based intervention to support their child’s development?

## 2. Materials and Methods

### 2.1. Study Design

This study represents qualitative thematic analysis, which used a convenience sample of caregivers who had accessed services at the community-based rehabilitation (CBR) centre in Albaqaa Refugee Camp in northern Amman, Jordan, from January to May 2023.

### 2.2. Participants and Recruitment

The CBR centre provided the principal investigator (R.A) with contact information of families receiving services at the centre. The inclusion criteria were that the participants must be refugees above the age of 18 and identify as a caregiver to one or more children with a disability, such as physical, cognitive, genetic, and/or developmental. It is important to note that for the purpose of this study, refugee status is defined by individuals who receive services through the refugee camp and live in this community. Those who do not meet the inclusion criteria stated were excluded from this study.

### 2.3. Survey Design

The study team developed a survey with open-ended questions in Arabic by incorporating themes from the previous literature regarding barriers and facilitators of community-based healthcare, as well as the previous literature regarding the use of MBIs with refugees [5,26,33,36,40,41].

The first part of the survey consisted of a seven-question demographic questionnaire developed to reflect the inclusion criteria. We asked the individuals to identify their refugee status, the presence of developmental disability and/or diagnosis in their child, the child’s age, the child’s exposure to traumatic events, and the presence of mental health services. The demographic questionnaire is displayed in Appendix A and Appendix B. The first of these sections was titled “Part One: Current Challenges with Caregivers and Children”. This section consisted of five questions that inquired on the child’s daily struggles, the supports caregivers currently utilise, and the barriers they face in accessing supports. The second was titled “Part Two: Mindfulness Strategies with Children”. After offering a short definition of MBIs, five questions were displayed for the caregivers to share their initial thoughts of using MBIs with their children and what barriers they could foresee in accessing or using MBIs. These sections are displayed in Appendix B. The full survey took up to 15–20 min to complete from start to finish.

### 2.4. Data Collection Procedures

The refugee caregivers’ contact information was accessed using the CBR Centre’s contact sheet. The survey was sent to by the principal author using WhatsApp on 6 March 2023 and was kept open for two weeks. The answers and data were collected using Google Sheets on an encrypted and password protected cloud-server that only the research study members had access to. They were translated from Arabic and transcribed into English by a blinded translator (M.F).

### 2.5. Ethical Considerations

Ethical approval for this study was granted on 11 February 2023 by the Mass General Brigham Institutional Review Board (Protocol #: 2022P000365). The caregivers were informed of the aims and potential risks of the study, and informed consent was obtained from them. This study was conducted following the principles of the Declaration of Helsinki. The collected data were secured in a password-protected and cloud-based server and de-identified during the coding process. The data will be destroyed after 10 years to ensure the safety of protected health information.

### 2.6. Data Analysis

This study utilised a content analysis approach in order to interpret the meaning of the qualitative data by developing codes and a coding scheme. First, the open-ended question responses were summarised into a table to begin the descriptive coding process. Second, the study team members reviewed the data for familiarisation by reading and re-reading to immerse themselves in the content. Third, initial coding was conducted using line-by-line analysis through identifying and labelling the key concepts emerging from the data. Fourth, categorisation by grouping similar codes in broader categories labelled as themes was completed. Finally, the codes and themes schema was reviewed and refined to ensure consistency and clarity. Once completed, a codebook using Google Sheets was developed, which included a title for each theme, a category, a code, and exemplars [42]. The demographic data were analysed using frequency and summary statistics.

To increase the reliability of the methods of analysis used in this study, member checking was used. Key participants reviewed the data interpretations, which helped confirm the accuracy and authenticity of the findings. The study team consisted of three doctoral candidates of OT at the MGH Institute of Health Professions. The principal author was one coder (Z.F), while the other two were contributors with experience in qualitative research.

Inter-coder consistency was also used to ensure coding reliability; multiple coders reviewed the data independently, with any discrepancies resolved through discussion until consensus was reached. The coders used emojis to organise the codes; one distinct emoji was assigned to each code to avoid misinterpretation. Conflicts and questions regarding the codes were resolved during team meetings by way of peer debriefing to increase reliability.

## 3. Results

Of the 156 individuals invited to participate, 28 refugee caregivers completed the survey, and 26 met the inclusion criteria and were eligible to be enrolled in the study. The demographic data are presented in Table 1. All but four participants were identified as refugees. As forementioned, they were still considered in this study as they live in a refugee host community and benefit from refugee camp services.

The diagnoses include Autism Spectrum Disorder, Rett’s syndrome, cerebral palsy, speech delays, and apraxia, among the other impairments noted.

Five themes emerged from the content analysis of the open-ended questions: (1) daily challenges; (2) Support and Strategies; (3) barriers to accessing support; (4) MBIs: Caregiver Perceptions; and (5) MBIs: Barriers. The development of the themes, categories, and codes is presented in Table 2.

Under the first theme ‘Daily Challenges’, several categories emerged, which describe the difficult daily experiences of caring for children with disabilities. These challenges may be innate, such as behavioural or communication-based. These challenges may also come as a result of a lack of accessible environments, which impede a child’s independence.

The caregivers described the support they are currently given, which was illustrated within the next theme: “Support and Strategies”. The parents described seeking support from therapists, physicians, friends, and family. It is important to note that this support targets physical and communication-based impairments, rather than emotional, behavioural, or psychological. The strategies to target emotional challenges were addressed in another category within this theme, where one parent described attempting to mitigate their child’s emotional challenges independently, or sought no help.

For the third theme ‘barriers to accessing support’, the majority of the categories described within this theme are logistic-based barriers, including financial; environmental; and those related to a lack of providers, time, or health restrictions. However, it is significant to note the recurrence of the word “embarrassment” being brought up as a barrier, which is categorically defined as “stigma” within the codebook. As for the other barriers mentioned, MBIs directly target the consequences of having little to no resources to expand a child’s scope of care.

This study suggests that MBIs can be free of charge, home-based, family-driven, and do not need to rely on the availability of health-care providers. For the fourth theme ‘MBIs: Caregiver Perceptions’, the caregivers who were previously exposed to the use of MBIs described their experiences within the category “previous use of MBIs”. Of these, several caregivers felt positively about these experiences, while the others did not feel that MBIs were sufficiently addressing their child’s needs. Otherwise, the caregivers who did not have any experience using MBIs with their children provided varied perceptions. The responses varied from “all of them are helpful for the child” to “she cannot do any of those”. Several caregivers brought up a pertinent thought: that MBIs are not currently being offered. This is reflected in the last theme ‘MBIs: Barriers’. As previously stated, the caregivers reflected on financial, environmental, and logistics-based concerns in evaluating the possibility of incorporating MBIs into their child’s care. Their concerns are important in considering further steps. Stigma was brought up again in referencing societal and cultural norms and the effects on mental health care.

## 4. Discussion

This study aimed to investigate the use of mindfulness-based interventions (MBIs) as a feasible, community-based, and accessible mental health strategy. All the caregivers were Arabic-speaking and of Arab ethnicities, which provides cultural context to understanding the stigma surrounding mental health care for refugee children with disabilities. The qualitative themes reveal persistent challenges that the refugee caregivers identified in their children. Such challenges include behaviour communication and other cognitive difficulties, all of which affect their child’s participation in daily life and their ability to access healthcare, e.g., not being able to get on a train or leave the house.

The caregivers reported feeling supported by community-healthcare professionals, such as physicians, therapists, and teachers. It is important to note that this support is all community-based and typically received in community-based rehabilitation centres in refugee camps. This emphasises the importance of community-based programmes in supporting refugee caregivers as the main reference point in their child’s care. Many community-based programmes are being dissolved worldwide, and this jeopardises the feeling of support caregivers with disabilities face in marginalised communities such as refugee camps. However, several caregivers reported keeping things to themselves and avoiding help from others to support their children. Avoidance coping strategies are also common among refugees and can lead to social isolation, as well as negative life outcomes [43].

Many respondents were concerned with their financial ability to obtain healthcare services. In addition, the caregivers noted environmental barriers such as transportation impeding their ability to access care, including whether they have reliable transport, or whether it is difficult to bring their child to a public system. The lack of accessible and appropriate mental health providers was reflected in the answers, and this trend has been recognised in the literature [5]. Finally, a notable barrier listed in the responses was the stigma. The caregivers cited “embarrassment” as limiting their participation in health care. As mentioned, cultural context is imperative in analysing these data, as mental health care and disability status are emerging and controversial topics among traditional Arab families. It is often more likely to seek support from religious communities or family circles, rather than opening up to people outside of entrusted communities. Even though the sample is small, all these themes confirm what has been observed across the literature as an absence of providers, financial instability, stigma towards mental health disorders, and a lack of mental health programmes in Jordan restricting access to mental health support [5,7,41,44].

When it comes to the caregivers’ perceptions of using MBIs with their children, the responses were positive. The caregivers were generally aware of MBIs; most did not have previous experience, while a few caregivers did. Some caregivers who had previous experience felt that they did not help their children. One caregiver with previous experience felt positively about the effect of MBIs on their child. A popular MBI of interest among caregivers was dance and movement. The other popular strategies included breathing strategies, followed by arts and crafts. Overall, the caregivers had positive perceptions of the potential use of MBIs with their children. However, the other caregivers were more concerned with obtaining other services, such as speech therapy, and conveyed negative perceptions of the potential of MBIs. Such responses characterise a general lack of awareness about the need for mental health care and the positive benefits of mindfulness among this community [7,43,44].

Although our sample size was small, the responses were varied and allowed us to identify the important themes that should be considered when evaluating and designing community-based mental health programmes for refugees. There is a gap in the literature on the barriers and challenges that refugee caregivers of children with disabilities face in accessing mental health care for their children. This study provides a foundation for the addition and expansion of accessible mental health care for refugee children with disabilities. Furthermore, given the literature and background information stated earlier, this study demonstrates further reasoning to support the use and applicability of MBIs as a means to address the barriers that caregivers face. Given the financial and mental health crises of this region, this paper provides families and community-based rehabilitation centres with a low-cost alternative to provide support and nurturing to refugee children with disabilities.

### 4.1. Study Limitations and Future Recommendations

This study is foundational, as it is the first of its kind to specifically explore refugee caregivers’ perceptions of using MBIs in the Arab region, an alternative, yet accessible mental health strategy. It is also the first study to explicitly cite and suggest the use of MBIs to support refugee children with disabilities. Our recruitment for this study was targeted, which allowed for a broad range of responses from a sample of refugee caregivers of children with disabilities or developmental delays. However, care should be taken when generalising the results of this study due to several limitations: its small study size and the participants. In addition, the gender of the children with disabilities was not listed. It would have been interesting to observe if there were differences in attitudes whether the individual was a male or a female.

For recruitment for this study, we used a convenience-based sampling technique, so the results only reflect the perceptions of a specific pool of refugees within the CBR centre who are caring for a child with a disability or impairment. Therefore, the sample size was not fully representative of other caregivers’ perceptions in the refugee community. For example, it is important to explore the perceptions of refugee caregivers who do not receive support from community centres. It would also be important to evaluate the perception of caregivers who report that their child is specifically facing mental health concerns, as this was not specifically addressed in this survey.

In addition, there were varying levels of reading comprehension among the caregivers, which was reflected in their responses; some provided one-two-word answers, while others wrote a few sentences. Some answers directly mirrored the question using the same language, which further emphasised the variability in reading comprehension levels. Given the nature of the survey, it was impossible to ask follow-up questions specific to the responses. This prevented the coders from appropriately reflecting all the participants’ perceptions of the codes, as ambiguous answers were not coded. However, the caregivers may not be conveying an accurate picture of their child’s current functioning because of self-report biases due to stigma or other variables. Finally, this study’s translator used a Lebanese dialect, which may be different from the dialect in which the caregivers write and speak. This could create further biases and misinterpretations that accurately reflect the caregivers’ perspectives.

Future studies should recruit from different sites and include specific demographic information that is essential to contextualising answers. These include the gender of the child, the number of children in the family, and specific demographic information about the caregivers. Although recruiting over WhatsApp was feasible and easy, recruitment in person may help to increase of sample size and variety of refugee participants.

### 4.2. Implications for Occupational Therapy Practice

Occupational therapy in this study was integral in considering the environmental and personal factors that determine the level of a refugee’s meaningful participation in mental health care. This provides the occupational therapist with a unique role in designing programmes that transcend barriers. Furthermore, occupational therapists incorporate MBI strategies into interventions to enhance functional participation in various populations [45]. Additionally, occupational therapists are uniquely equipped to work with the refugee population [46]. Therefore, this study provides opportunities for occupational therapy to be involved in the research and the development of an MBI programme for refugees.

## 5. Conclusions

This foundational study on the application of mindfulness-based interventions among Arab refugee children with disabilities suggests that this strategy could be acceptable and helpful to children with disabilities. This is the first study to explore the perspectives of caregivers of MBIs. This study adds to the existing literature by considering the specific personal factors and contexts that affect participation in mental health care and shape perceptions regarding MBIs, especially among marginalised groups like forced migrants with disabilities. Designing a programme for this community requires cultural considerations and caregivers’ input to ensure the appropriateness of services in the context of a refugee’s life. By considering our findings, planning to integrate MBIs in mental health programming would work towards transcending some of the identified barriers that caregivers can accept and utilise in either community or home settings to improve their child’s lives.

## Figures and Tables

**Table 1:** Participants sociodemographic characteristics.

Sample Characteristics	*N* = 26	%
Child’s Age, y (Mean; SD)	8.23 ± 4.21	
0–2 years	1	3.8%
3–5 years	9	34.6%
6–7 years	3	11.5%
8–10 years	6	23.1%
11–13 years	2	7.7%
14–16 years	3	11.5%
17–19 years	1	3.8%
Not stated	1	3.8%
Refugee Status		
Identifies as refugee	22	84.6%
Does not identify as refugee	4	15.4%
Child’s Diagnosis		
Down Syndrome	3	11.1%
Cerebral Palsy	2	7.4%
Speech Delay	6	22.2%
Hearing Deficit	3	11.1%
Autism Spectrum Disorder	4	14.8%
Rhett’s Syndrome	1	3.7%
Other Cognitive	8	29.6%
Exposure to Trauma		
Death in Family	1	3.8%
Displacement	1	3.8%
Violence	3	11.5%
Illness	4	15.4%
None/Others	17	65.4%
Mental Health Services		
Child does receive services	6	23.1%
Child does not receive services	20	76.9%

**Table 2:** Development of codes, categories, and themes.

Theme	Category	Code
Daily Challenges	Behavioural and Emotional	“[There is an] excessive demand for food, I feel like it’s an emotional eating.” “Nervousness and mood swings.” “Screaming and hyperactivity.”
	Communication	“He cannot express what he wants by words.” “[I don’t know] how to please him.” “The child has difficulty in speaking, which leads to difficulty in learning and interacting.”
	Lack of Independence	“She cannot stand or walk and needs her parents to carry her around… She does not understand the dangers around her.” “Inability to perform his needs alone [is a challenge].”
	Environmental	“[There are challenges] in the living situation.” “The environment [is a daily challenge].”
	Cognitive	“Behavioral and cognitive challenges.” “Difficulty with speech and learning.”
Support and Strategies	Therapies	“He was getting speech therapy at the community social center of the area” “… as well as two physical and occupational therapists.”
	Speak with a Physician	“Yes, we are following up with a neurologist.” “I spoke with the physician, but there are still developmental problems.”
	Speak with friends/family	“I talked to my family, and they supported me a lot.” “…a teacher to develop the growth and skills that my child needs.”
	Support from the Centre	“[We overcome these challenges] through the community rehabilitation center, the teacher.”
	Parent-Driven Strategies	“As for the speech: I ask him to repeat the conversation and clarification. As for the [emotional] eating I try to occupy him with another activity, but he only responds sometimes.”
	No Support	“I keep things to myself.” “No [strategies].”
Barriers to Accessing Supports	Financial	“The cost and financial situation [prevents me from getting the support].” “I cannot afford to go to a different clinic.”
	Environmental	“Being in the third floor is difficult, and transportation also.” “Accessibility [is a barrier].” “Transportation is difficult.” “Difficulty controlling my son in public transport.”
	Stigma	“It is embarrassing to get support.” “Embarrassment because she cannot use the restroom.” “Embarrassment [is a barrier].”
	Health restrictions	“Health [is a barrier].”
	Time	“Time [is a barrier].”
	Lack of Providers	“There is no support in the center.” “For psychological support, there is no appropriate specialist.”
MBIs: Caregiver Perceptions	Previous use of MBIs	“Yes, like meditation, physical activity and arts.” “[Yes], from the teacher’s guidance.” “I used meditation and physical activity, and I was able to adapt to my son’s situation.”
	No previous use of MBIs	“There is no strategy.” “I did not.”
	Positive perception of MBIs	“It will give my son more confidence in himself.” “It teaches me patience and calm.” Helpful for the mind, the body and the communication.” “It can help the child to express his feelings.”
	Negative perception of MBIs	“Helpful, but not enough to improve my son’s skills.” “Not useful, because he needs speech therapy.” “There is no response or improvement noticed.”
	Not sure how to feel about MBIs	“I do not know.” “It was not offered to me yet.”
	MBIs are not offered in community settings	“I did not get a strategy from anyone.” “There is none.”
	MBI that would be most helpful	“I prefer movement.” “Maybe breathing and crafts are a start.” “All of them are helpful for the child.”
	MBI is not applicable to the child	“She cannot do any of those.”
MBIs: Barriers	Financial	“Financial obstacles.” “Thank God, I have no obstacles except the financial. (…) The financial plays a big role even in health problems.”
	Psychosocial	“Family problems” “He does not respond to me because he is stubborn.” “Psychological and social [barriers].”
	Stigma	“The society thinking that he is handicapped.”
	Environmental	“There is no support to go to the place where she gets help. She cannot walk or speak.” “There is no place to do these interventions.” “Cost and transportation [are barriers].”
	Logistical	“[I am busy] taking care of the children, helping them study and household chores.” “The appropriate time and duration of the strategies [are barriers].” “Living difficulties [are barriers].”

## Data Availability

The data supporting the findings of this study are available upon request from the first author [ZF]. The data are not publicly available due to restrictions, e.g., they contain information that could compromise the privacy of the research participants.

## Appendix A. Demographic Survey

1. Are you over 18?
2. Do you identify yourself as a refugee?
3. Are you caring for a child with a developmental disability? Developmental disabilities include attention deficit hyperactivity disorder, autism spectrum disorder, cerebral palsy, hearing loss, intellectual disability, learning disability, and visual impairment.
4. Does your child have a formal diagnosis? If so, what is the diagnosis?
5. How old is the child you are caring for?
6. Has your child been exposed to any of the following?
  - War
  - Disasters/Explosions
  - Displacement
  - Serious injury or Illness
  - Watching violence
  - Loss of a family member/caregiver/friend
  - None of the Above
  - A different form of shock that is not listed: _____
7. Is the child currently receiving mental health services?

## Appendix B. Survey Questions

**Part One: Current Challenges with Caregivers and Children**

1. What challenges do you face throughout the day with your child(ren)? Think about the emotional or behavioral challenges you face throughout the day with your child.
2. How have you overcome these challenges in the past? For example, do you talk to a doctor, family member, or friend? Do you keep things to yourself?
3. What support is available for your child(ren)? Is it effective? Think about things like social support, family support and emotional support.
4. What support do you have in place for you? Are they working? For example: social support, family support, emotional support.
5. What is preventing you from getting the support you and your child(ren) need? Think about barriers such as cost, location, transportation, accessibility, embarrassment, time, etc.

**Part Two: Mindfulness Strategies with Children**

*Mindfulness is an umbrella term used to describe different coping strategies, such as: meditation, yoga, arts and crafts, or physical movement. Mindfulness-based strategies promote non-judgmental thinking, mind-body connection and increased awareness of the present moment to support the development of self-compassion and coping skills.*

1. Have you or your child(ren) ever used Mindfulness-based strategies to help overcome challenges mentioned above? If so, what types have you used and how have they affected you?
2. Why do you think mindfulness-based strategies would be a helpful coping strategy for your child(ren)? If not, how come?
3. What type of mindfulness-based strategy do you think would be most effective for your child(ren)? Art-based, dance and movement, meditation, deep-breathing, etc.
4. What barriers do you anticipate would prevent you from using mindfulness-based strategies with your child(ren)?
